# Thymosin Beta-4 Induces Mouse Hair Growth

**DOI:** 10.1371/journal.pone.0130040

**Published:** 2015-06-17

**Authors:** Xiaoyu Gao, Hao Liang, Fang Hou, Zhipeng Zhang, Mingtu Nuo, Xudong Guo, Dongjun Liu

**Affiliations:** National Research Center for Animal Transgenic Biotechnology, Inner Mongolia University, Hohhot, Inner Mongolia, China; Vanderbilt University, UNITED STATES

## Abstract

Thymosin beta-4 (Tβ4) is known to induce hair growth and hair follicle (HF) development; however, its mechanism of action is unknown. We generated mice that overexpressed Tβ4 in the epidermis, as well as Tβ4 global knockout mice, to study the role of Tβ4 in HF development and explore the mechanism of Tβ4 on hair growth. To study Tβ4 function, we depilated control and experimental mice and made tissue sections stained with hematoxylin and eosin (H&E). To explore the effect of Tβ4 on hair growth and HF development, the mRNA and protein levels of Tβ4 and VEGF were detected by real-time PCR and western blotting in control and experimental mice. Protein expression levels and the phosphorylation of P38, ERK and AKT were also examined by western blotting. The results of depilation indicated that hair re-growth was faster in Tβ4-overexpressing mice, but slower in knockout mice. Histological examination revealed that Tβ4-overexpressing mice had a higher number of hair shafts and HFs clustered together to form groups, while the HFs of control mice and knockout mice were separate. Hair shafts in knockout mice were significantly reduced in number compared with control mice. Increased Tβ4 expression at the mRNA and protein levels was confirmed in Tβ4-overexpressing mice, which also had increased VEGF expression. On the other hand, knockout mice had reduced levels of VEGF expression. Mechanistically, Tβ4-overexpressing mice showed increased protein expression levels and phosphorylation of P38, ERK and AKT, whereas knockout mice had decreased levels of both expression and phosphorylation of these proteins. Tβ4 appears to regulate P38/ERK/AKT signaling via its effect on VEGF expression, with a resultant effect on the speed of hair growth, the pattern of HFs and the number of hair shafts.

## Introduction

Thymosin beta-4 (Tβ4) is a 4.9-kDa actin-sequestering peptide that contains 43 amino acids [1]. Tβ4 plays an important role in cell motility [2,3], and has multiple biological activities, such as promoting angiogenesis [4,5], wound healing [4], tumor metastasis [6,7], hair growth [8,9] and anti-apoptosis [10]. It has been reported in rats and mice that exogenous Tβ4 plays an important role in wound healing and hair growth [11]. When studying the influence of Tβ4 on wound healing, Philp et al. accidentally found that hair grew more rapidly around the edges of wounds. It was subsequently shown that Tβ4 induced rapid hair growth on the dorsal skin of athymic mice and depilated regions of rats and mice. Similarly, rat whiskers grown in vitro grow rapidly when treated with Tβ4 [9]. When examining the distribution of endogenous Tβ4 expression during the sequential phases of depilation-induced hair growth, high levels of Tβ4 expression were observed in the developing hair follicles (HFs). Additionally, Tβ4 expression is also high in the developing HFs of mice and clonogenic mice HF keratinocytes cultured in vitro for 7–10 days [9,12]. The generation of Tβ4 transgenic mice confirmed that Tβ4 promotes hair growth [12].

While it is apparent that Tβ4 plays a role in hair growth and its molecular mechanism is unknown. To explore the role of Tβ4 in HF development, we generated mice that overexpress Tβ4 in the epidermis (KTP mice) and Tβ4 global knockout mice (KO mice). Mechanistically, it has been shown that Tβ4 accelerates angiogenesis by increasing vascular endothelial growth factor (VEGF) expression [13], which may be secondary to the effects of Tβ4 on MAPK, PKC and WNT signaling [14]. Thus, we also explored the relationship between Tβ4 and VEGF expression in the skin, and dissected the underlying signaling pathways associated with this effect.

## Materials and Methods

### Ethics statement

All mice surgeries were performed under 2,2,2-tribromoethanol (Sigma-Aldrich, St Louis, MO) anesthesia. All studies adhered to procedures consistent with the National Research Council Guide for the Care and Use of Laboratory Animals, and were approved by the Institutional Animal Care and Use Committee of the Inner Mongolia University.

### Construction of KTP overexpression plasmid

The KTP overexpression plasmid contained a 2.8-kb fragment that comprised the keratin14 (K14) promoter (2,350 bp) from humans, the full-length mouse Tβ4 cDNA (135 bp) and a 3' polyA signal sequence (225 bp) from BGH. The K14 promoter (accession number: U11076.1) was cloned by PCR and inserted into a pMD19T vector (TaKaRa, Tokyo, Japan) with a T-A connection, named pMD19T-K. Mouse Tβ4 cDNA sequence (accession number: NM_021278.2) was cloned, cut using *Xba*I and *Kpn*I and inserted into the pMD19T-K vector. The 3' polyA signal sequence was cut using *Kpn*I and *Eco*RI and inserted into the pMD19T-KT vector. The resultant cassette named KTP was cut with *Eco*RI and *Hind*III and isolated by agarose gel electrophoresis. The KTP band was then isolated from the agarose gel, purified and used for pronuclear injection.

### Generation of Tβ4 knockouts using TALEN

**Fig 1:**
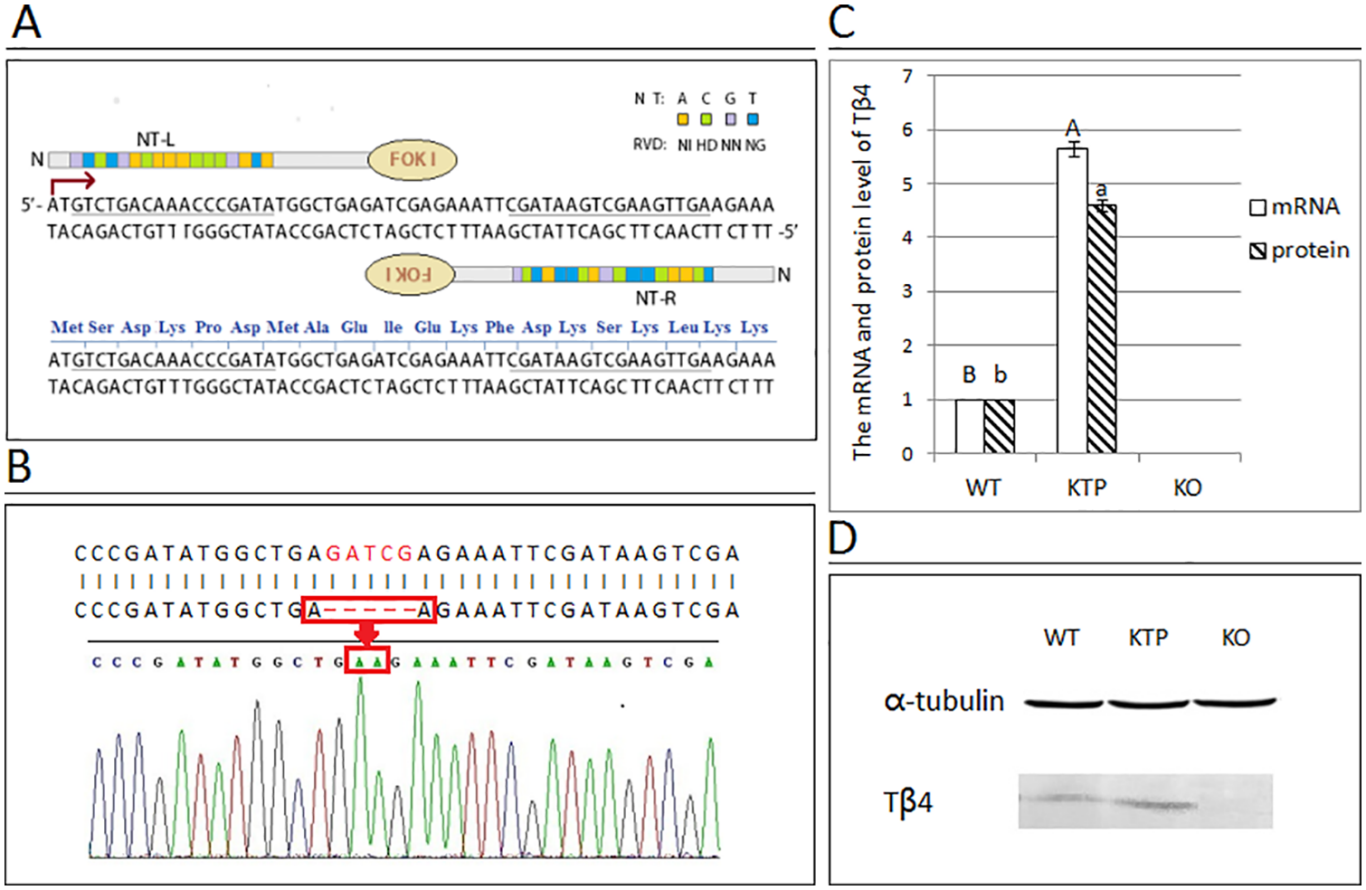
Construct design and strategies for Tβ4 knockout mice by TALEN and an analysis of the transcriptional and protein expression levels of Tβ4. (A) A representative schematic of the TALEN is indicated, showing the left and right TALEN DNA recognition sequences (underlined) and the spacer region fused to the FokI endonuclease catalytic domain. (B) The red box indicates the gap in the genomic DNA of KO mouse. (C) This graphical representation demonstrates the pattern of Tβ4 mRNA and protein levels between the dorsal skin samples of the three groups obtained from real-time PCR and western blotting analysis. Gapdh in real-time PCR and α-tubulin in western blotting were used as internal controls. Samples from the WT group were normalized to 1. Each bar represents the mean ± SEM (n = 3). Different superscripts on the bar indicate statistical difference (P < 0.01). (D) Protein expression of Tβ4 was detected by western blotting. α-Tubulin was used as the loading control. The molecular mass of Tβ4 and α-tubulin were approximately 4.9 kDa and 50 kDa, respectively. The results of one representative image of three independent experiments are presented.

We used TALENs to disrupt the Tβ4 gene, designing a spacer nearby the ATG start codon of Tβ4 to generate a frame shift mutation and loss of Tβ4 function. The appropriate TALEN targeting site was designed using the online TAL Effector Nucleotide Targeter 2.0 software program (https://tale-nt.cac.cornell.edu/node/add/talen-old) (Fig 1A). The targeted sequence was within the second exon of Tβ4 (accession number: NC_000086), on chromosome X. DNA recognized sequences were as follows: left: 5’-GTCTGACAAACCCGATA-3’, right: 5’-TCAACTTCGACTTATCG-3’. The construction of the Tβ4 targeted TALENs was conducted according to the protocol of the FastTALETM TALEN Kit (Sidansai Biotechnology, Shanghai, China). The Tβ4-targeted TALENs RNA mixture was microinjected into zygotes to obtain Tβ4 KO mice, as described below.

### Microinjection and genotyping

TALEN RNAs were prepared using the MEGAscriptH SP6 Kit (Life Technologies, Carlsbad, CA) in vitro, and purified using an RNeasy Mini Kit (Qiagen, Dusseldorf, Germany) according to the manufacturer’s instructions. The RNA concentration was measured using a NanoDrop 2000C spectrophotometer (Thermo Scientific, Rockford, IL) and diluted to obtain a working concentration of 50 ng /μl with the injection buffer supplied in the FastTALETM TALEN Kit (Sidansai Biotechnology). For gene targeting, zygotes from C57BL/6 females were injected with a Tβ4-targeted TALENs RNA mixture. Microinjection was performed using a micromanipulator system (Nikon, Tokyo, Japan). Injected zygotes were cultured in KSOM medium (Millipore, Bedford, MA) at 37° and then transferred to pseudo-pregnant ICR female mice at 0.5 dpc. The KTP fragment described above was directly microinjected as described above.

In order to confirm the successful generation of KTP and KO mice, genomic DNA was extracted from the tail tips of newborn mice using a TIANamp Genomic DNA Kit (Tiangen Biotech, Beijing, China). For genotyping of Tβ4 KO mice and KTP mice, PCR was performed using the following primers: KO primers, F: 5’- AGGACTCGACAAGGAAAGCACACAG -3’, R: 5’- GCTGCCCCTCCCTTC CTCCT CCCGG -3’; and KTP primers, F: 5’-GCCTGTCTGTGCCCAAGGTGA -3’, R: 5’- ACGGGG GAGGG GCAAACAAC -3’. PCR products of ungenotyped KTP mice were separated by agarose gel electrophoresis to observe the expected bands, and PCR products of ungenotyped KO mice were used as templates for DNA sequencing. The DNA sequence data of KO mice were compared to the Tβ4 mRNA reference sequence.

### Hair growth analysis

HFs are present during the telogen phase for mice aged 6–8 weeks. It is known that depilation induces synchronization of HFs starting from the telogen phase [15]. Thus, the speed of hair growth was observed in wild type (WT), KTP and KO mice aged 8–10 weeks. They were depilated on the dorsal skin with a mixture of rosin and paraffin wax. Hair growth in all mice was monitored for 11 days after depilation, at which time photographs were taken. Three mice were used for each group and the experiment was repeated three times.

### Histology of HF development

Dorsal skin tissue samples from the three groups were collected during the same hair growth conditions (WT mice: 13 days; KTP mice: 11 days; KO mice: 16 days) after depilation, fixed in 4% formaldehyde at 4°C and embedded in paraffin blocks to obtain 7-μm transverse sections that were stained with hematoxylin and eosin (H&E). The pattern of HFs was then observed and the number of hair shafts was counted. Digital photomicrographs were taken from representative areas at a fixed magnification of 200x. Three mice were used in each group and the experiment was repeated three times. The surplus skin samples were stored at −80°C prior to mRNA and protein extraction.

### Real-time PCR analyses

Analysis of Tβ4 and VEGF mRNA levels was conducted using real-time quantitative PCR and the ΔΔCT method. RNA from dorsal skin tissue samples was extracted using an RNAiso plus kit (TaKaRa), according to the manufacturer’s instruction. Isolated RNA was transcribed into cDNA using a PrimeScript RT reagent Kit with gDNA Eraser (TaKaRa). The resultant cDNA was then used as a template to detect mRNA levels of Tβ4 and VEGF. Gapdh was used as the internal control. Real-time PCR was performed using the following primers: Gapdh primers, F: 5’-TGTGTCCGTCGTGGATCTG A -3’, R: 5’-CAACACCTCAACAGGAGTGGACA -3’; Tβ4 primers, F: 5’-AAACCCGATATGGCTG AGATTG -3’, R: 5’-GCCTGCTTGCTTCTCCTGTT -3’; and VEGF primers, F: 5’-CATTGAGACCCT GGTGGA CAT -3’, R: 5’-CTGGCTTTGGTGAGGTTTGAT -3’.

### Western blotting

Whole protein extracts of dorsal skin tissue samples were collected and quantified using a Bradford assay. Primary antibodies against α-tubulin, Tβ4, VEGF, P38, ERK1/2 and AKT (Abcam, Cambridge, MA) were respectively diluted to 1:1,000 with 0.05% non-fat milk in TBST. Rabbit anti-phospho (p)-P38, p-ERK1/2 and p-AKT (Cell Signaling Technology, Danvers, MA) were diluted to 1:1,000 with 0.05% bovine serum albumin (BSA; Sigma-Aldrich). A secondary antibody (anti-rabbit IgG; Abcam) was diluted to 1:10,000 with 0.5% non-fat milk or BAS in TBST to reveal primary antibody binding. The expression of α-tubulin was used as an internal loading control and the optical density of protein bands were measured using Image J software (NIH, USA).

### Statistical analysis

Statistical analyses of the number of HFs, real-time PCR and western blotting results were conducted using analysis of variance (ANOVA). Differences among the three groups of mice were evaluated using Duncan's multiple comparison tests. Three mice were tested in each group. All data from more than three repeated experiments were expressed as the mean ± SEM and a difference of P < 0.01 was considered significant.

## Results

### Generation of and Tβ4 expression assays in Tβ4-overexpressing mice and global knockout mice

From the resultant offspring, three KTP mice and eight KO mice were identified by PCR and sequencing (Table 1). The expected band appeared on agarose gels after PCR assays of the samples from KTP mice. After two crosses, homozygous KO mice were born and their genotype was confirmed by PCR and sequencing. A five base gap was detected in the genome of KO mice (Fig 1B). Based on these findings, we concluded that KTP mice and KO mice were successfully generated. The overexpression and deletion of Tβ4 were confirmed by real-time PCR and Western blotting analysis with the dorsal skin samples of WT mice, KTP mice and KO mice. Relative to WT mice, Tβ4 mRNA expression was 5.65 times higher in KTP mice (P < 0.01; Fig 1C). With regards to protein expression, Tβ4 was increased by approximately 4.6 times in KTP mice relative to WT mice, and no expression was detected in KO mice (Fig 1D).

**Table 1:** Construction of KTP mice and KO mice by microinjection.

Type	Transferred Embryos (Recipients)	Newborns (Birth Rate)	Founders (Mutation Rate)
KO	127(5)	25(19.7%)	8(32%)
KTP	225 (7)	40(17.8%)	3(7.5%)

### Tβ4 plays an important role in HF development and hair growth

**Fig 2:**
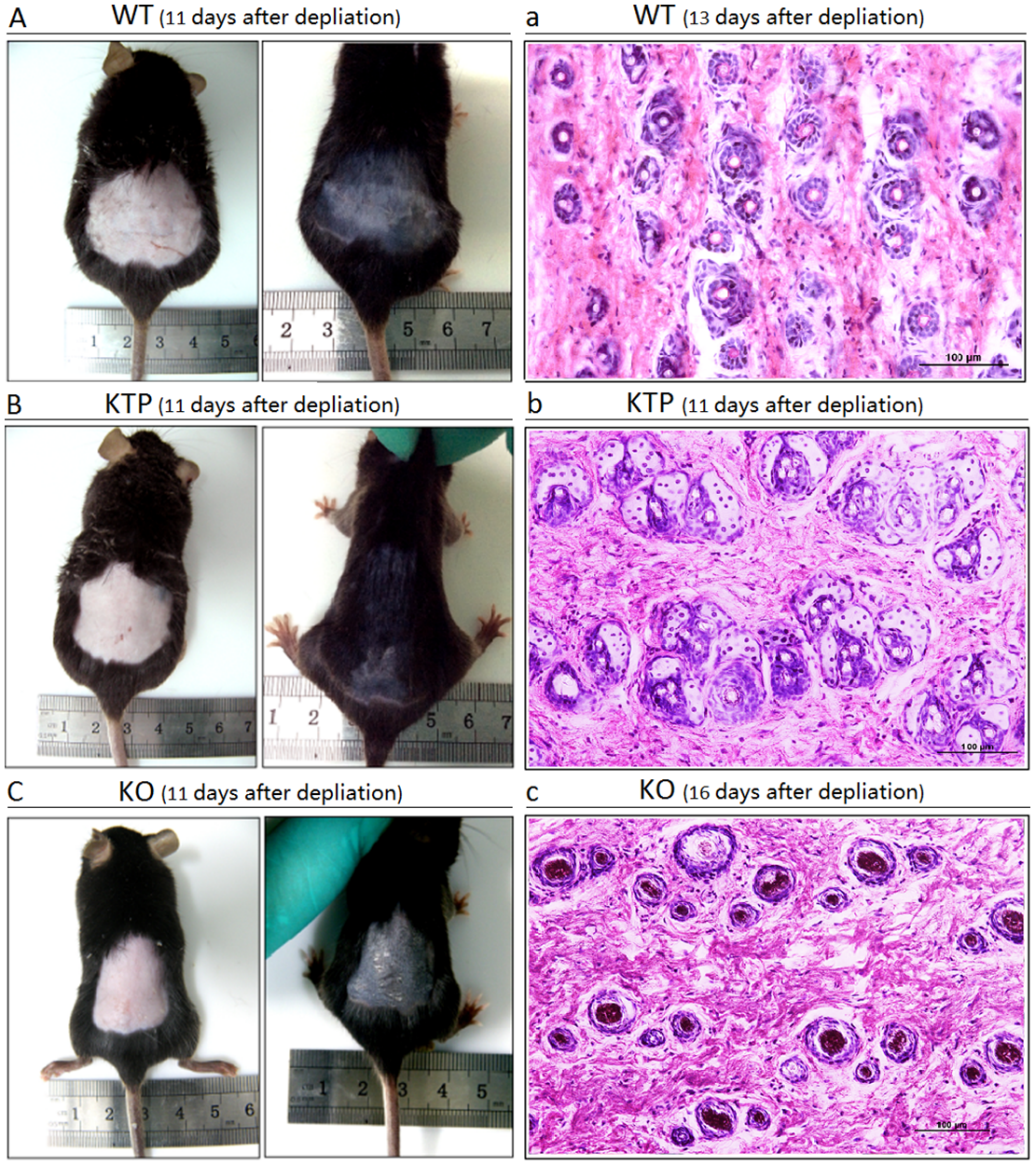
The morphological and histological observations of depilated mice. The conditions of hair re-growth were observed in WT (A), KTP (B) and KO (C) mice aged 8–10 weeks for 11 days after depilation. Cross-sections were stained with H&E to observe the pattern of HFs in WT (a), KTP (b) and KO (c) mice. The scale bar is 100 μm.

WT, KTP and KO mice were depilated at approximately weeks 8–10 of age. Eleven days after depilation, we observed that the hair of KTP mice was longer and thicker than that of WT mice (Fig 2A and 2B), whereas the depilated skin of KO mice became black without hair growth (Fig 2C). If the speed of hair re-growth in WT mice was considered to be normal, then KTP mice had faster hair growth, whereas this process was much slower in KO mice.

**Fig 3:**
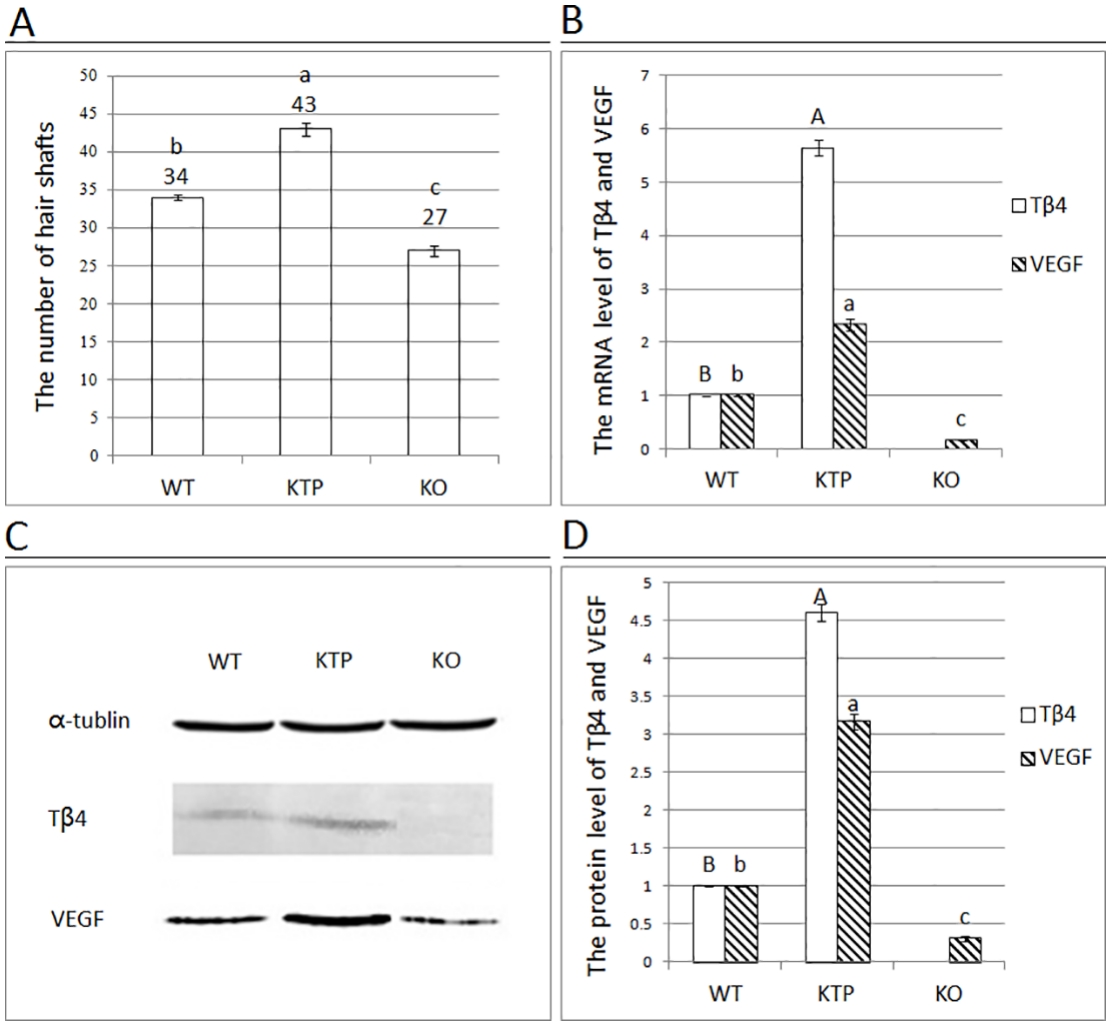
The number of hair shafts and analysis of the transcriptional and protein expression levels of VEGF. (A) Under x200 magnification, the number of hair shafts in the three groups of mice was counted in each field. Graphical representation demonstrating the number of hair shafts between the dorsal skin samples of the three groups obtained from cross-sections stained with H&E. (B) The mRNA levels of VEGF were examined in the three groups of mice by real-time PCR. (C) The results of western blotting assays. α-Tubulin was used as the loading control. The molecular mass of VEGF was approximately 45 kDa. (D) The protein expression levels of VEGF were analyzed by Image J software. In the above figures, the results of one representative image of three independent experiments are presented. Each bar represents the mean ± SEM (n = 3). Different superscripts on the bar indicate a statistically significant difference (P < 0.01).

By observing hair re-growth, we found that it was required 13 days in WT mice, 11 days in KTP mice and 16 days in KO mice to reach the same extent. Histological examination of dorsal skin samples was performed during the same hair growth conditions (WT mice: 13 days; KTP mice: 11 days; KO mice: 16 days) after depilation. H&E staining of tissue sections indicated that HFs of WT mice grew separately (Fig 2a), whereas HFs of KTP mice grew together and formed clusters (Fig 2b), and HFs of KO mice grew wantonly with no regularity (Fig 2c). By counting the number of hair shafts, we found that KTP mice had a significantly higher number of hair shafts compared to control mice, whereas the number of hair shafts in KO mice was reduced (Fig 3A).

### The expression level of Tβ4 correlates with VEGF expression

In order to assess the effect of Tβ4 overexpression or deletion on VEGF expression, we measured the mRNA and protein levels of VEGF in the skin samples of three groups. VEGF mRNA expression was 2.33 times higher in KTP mice compared to controls, compared to an increase of only 0.16-fold of control in KO mice (P < 0.01; Fig 3B). The protein expression level of VEGF was increased 3.17 times in KTP mice and was suppressed to 0.31-times the WT level in KO mice (P < 0.01; Fig 3C and 3D). The changes in VEGF expression showed the same trend to that of Tβ4 expression in WT, KTP and KO mice.

### The effect of Tβ4 on the MAPK signaling pathway

**Fig 4:**
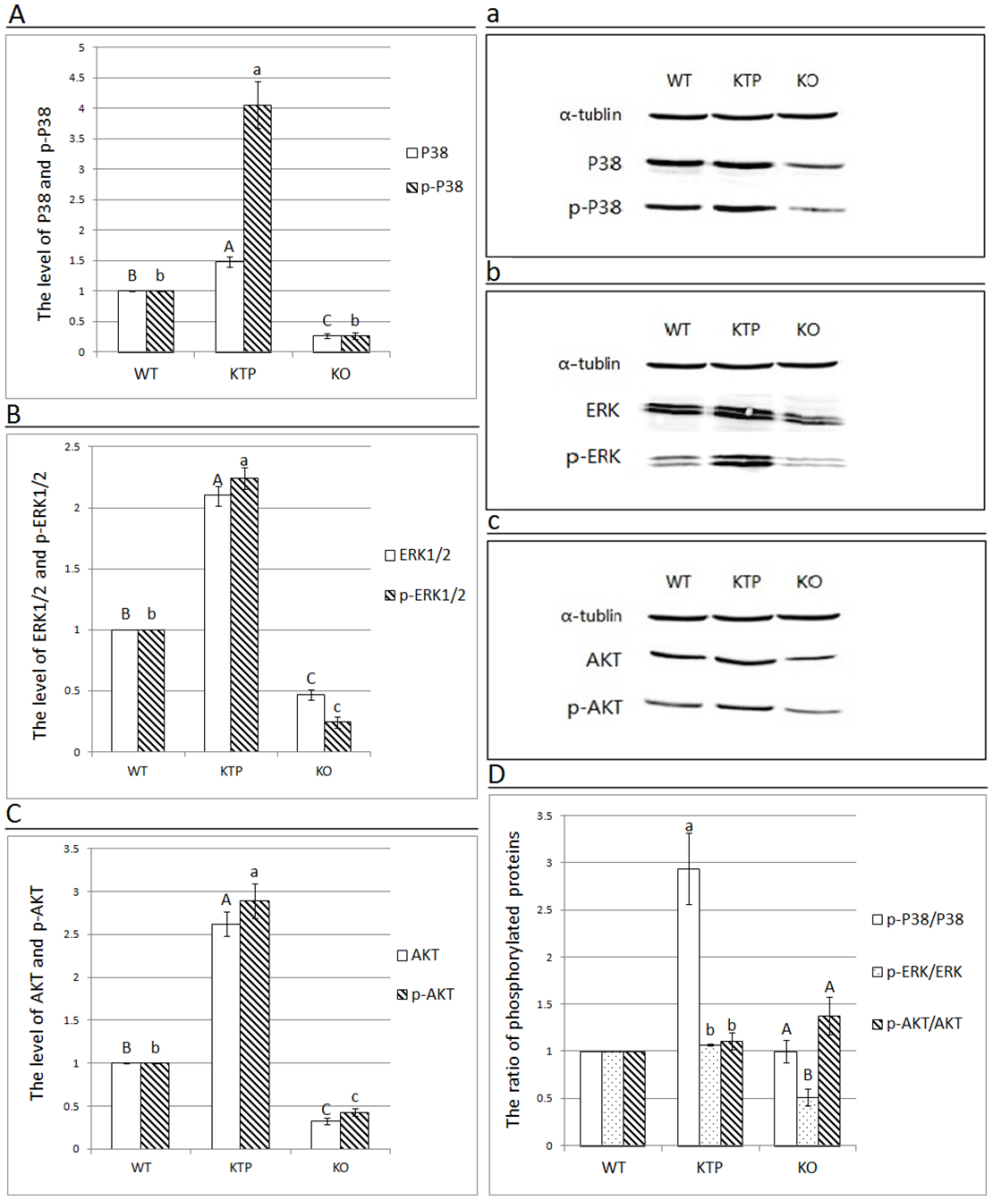
The protein levels of P38, p-P38, ERK1/2, p-ERK1/2, AKT and p-AKT and the ratio of phosphorylated proteins in the corresponding proteins. (a-c) The protein levels of P38, p-P38, ERK1/2, p-ERK1/2, AKT and p-AKT are shown. α-Tubulin was used as the loading control. The molecular masses of P38 or p-P38, ERK1/2 or p-ERK1/2 and AKT or p-AKT were approximately 38 kDa, 42/44 kDa, 56 kDa, respectively. (A-C) The protein expression levels of these proteins were then analyzed by Image J software. Graphical representation demonstrating the protein expression and phosphorylation levels between the dorsal skin samples of the three groups obtained from western blotting assays. (D) The ratio of phosphorylated proteins is shown as 1 in WT mice. In the above figures, the results of one representative experiment of three independent experiments are presented. Each bar represents the mean ± SEM (n = 3). Different superscripts on the bar indicate a statistically significant difference (P < 0.01).

In order to better understand the mechanistic link between Tβ4 and VEGF, we examined the expression levels of various different components of the MAPK signaling pathway in the dorsal skin of our experimental mice. Our results indicated that P38 and p-P38 were both significantly increased in KTP mice compared to WT mice (P < 0.01; Fig 4A and 4a). On the other hand, P38 expression was significantly reduced in KO vs. WT mice (P < 0.01); however, the level of p-P38 was also reduced and was not significantly different compared with WT mice (P> 0.05). With respect to ERK, the levels of ERK1/2 and p-ERK1/2 were significantly increased in KTP mice compared to WT mice (P < 0.01), whereas the expression of ERK1/2 and p-ERK1/2 were significantly decreased in KO mice (P < 0.01; Fig 4B and 4b).

### The effect of Tβ4 on the P13K signaling pathway

In order to better understand the mechanistic link between Tβ4 and VEGF, we examined the expression level of the PI3K signaling pathway in the dorsal skin of the experimental mice. We observed that the protein expression level of AKT was significantly increased in KTP mice (P < 0.01), but significantly decreased in KO mice compared to WT mice (P < 0.01; Fig 4C and 4c). With regards to p-AKT, its levels were significantly increased in KTP mice (P < 0.01), but not significantly different from WT in KO mice (P > 0.05). Finally, we calculated the ratio of phosphorylated proteins in each pathway and compared them between genotypes. In KTP mice, the ratio of p-P38/P38 was significantly higher (P < 0.01), while the ratio of p-AKT/AKT and p-ERK/ERK was approximately 1 (Fig 4D). In KO mice, the ratio of p-ERK/ERK was significantly reduced (P < 0.01), whereas the ratio of p-P38/P38 and p-AKT/AKT was approximately 1.

## Discussion

The results from the depilated WT mice in our study indicated that their characterization of hair re-growth was similar to normal depilated C57BL/6 mice during different phases [16,17]. Consistent with previous descriptions of transgenic Tβ4 mice created by Cha et al. [12], KTP mice generated in this study also had faster rates of hair growth than WT mice; in addition, the pattern of HFs were altered in KTP mice. The pattern of HFs in KTP mice has been observed in sheep and other animals [18], but not in normal mice. Tβ4 is known to play a key role in many physiological processes that are involved in early embryonic development and cardiovasculogenesis; it is therefore considered to be indispensable in murine growth and development. Recent studies show that healthy mice are provided by the conditional knockouts of Tβ4 and the global knockout of Tβ4 [19,20]. Here, the depilation result of KO mice showed that hair re-growth was slower than in WT mice and KO mice had fewer HFs. Therefore, Tβ4 was not required for either hair growth or HF development, but it did influence the number of hair shafts, change the pattern of HFs and accelerate hair growth.

It is well known that Tβ4 and VEGF play an important role in hair development, angiogenesis and recovery from cardiac injury [4,5,21], and it has previously been shown that Tβ4 induces VEGF expression [22]. Given this association, we studied VEGF expression levels in our experimental mice. Consistent with the literature, VEGF expression was linked with Tβ4 expression, such that in Tβ4-overexpressing mice, VEGF expression was increased and, in Tβ4 KO mice, VEGF expression was reduced. We interpreted this result to indicate that Tβ4 acts upstream of VEGF and can regulate its expression. VEGF is of major importance as an angiogenic factor for skin vascularization and plays an important role in the control of perifollicular vascularization and hair size during hair cycling [21]. Our data indicate that Tβ4 promotes hair growth by stimulating the expression of VEGF, which in turn induces angiogenesis around HFs and thereby regulates the speed of hair growth, the number of hair shafts and the pattern of HFs.

The downstream mechanism of Tβ4 signaling on VEGF and how it regulates hair growth and development is unknown. VEGF is also known as the vascular permeability factor [23], and plays a key role of angiopoiesis and micro-vascular permeability [24,25], acting through transmembrane protein receptors in endothelial cells and other cells to regulate transmembrane signal transduction [26]. One of the receptors that VEGF binds to is VEGFr-2. Previously, VEGF165 was shown to increase the phosphorylation of ERK, C-JUN and P38 via VEGFr-2 and thereby induced angiogenesis and HF development in humans [27]. It has also been shown that VEGF induces the proliferation of vascular endothelial cells via ERK1/2 and P38 [28,29,30,31]. The above results indicate that there is a close relationship between VEGF, P38 and ERK. P38 and ERK are two important proteins in the MAPK pathway, and AKT/PI3K has been associated with hair growth [32,33]. Thus, P38, ERK and AKT were chosen to assess the activation of P38/MAPK, ERK /MAPK and AKT/PI3K pathways in order to ascertain whether there is a relationship between Tβ4 and these signaling pathways. In KTP mice, we observed increased VEGF expression in connection with increased the phosphorylation of P38, AKT and ERK1/2. Conversely, in KO mice, VEGF expression was decreased, which resulted in reduced levels of ERK1/2 phosphorylation. These data are consistent with studies in human dermal papilla cells, which reported that the phosphorylation of ERK1/2 was changed in terms of intensity and the phosphorylation of P38, JNK and AKT was fixed in dermal papilla cells treated with VEGF or VEGFr-2 neutralizing antibodies [34].

In conclusion, we have identified an important role for Tβ4 in hair growth. We have clearly demonstrated that Tβ4 overexpression promotes hair growth, while the loss of Tβ4 suppresses it. In this study, Tβ4 was shown to stimulate the expression of VEGF, which may stimulate a downstream signaling pathway via the activation of the MAPK/P38, MAPK/ERK and PI3K/AKT signaling pathways. These studies establish the basis for the further exploration of Tβ4 function and its molecular mechanisms in hair growth. Furthermore, as with PTHrP [35], clinical studies need to be performed to assess its possible role in the treatment and prevention of hair loss.
